# Adverse birth outcomes among deliveries at Gondar University Hospital, Northwest Ethiopia

**DOI:** 10.1186/1471-2393-14-90

**Published:** 2014-02-27

**Authors:** Akilew Awoke Adane, Tadesse Awoke Ayele, Leta Gedefaw Ararsa, Bikes Destaw Bitew, Berihun Megabiaw Zeleke

**Affiliations:** 1 Department of Epidemiology and Biostatistics, Institute of Public Health, College of Medicine and Health Sciences, University of Gondar, Gondar, Ethiopia; 2 Department of Gynecology & Obstetrics, School of Medicine, College of Medicine and Health Sciences, University of Gondar, Gondar, Ethiopia; 3 Department of Environmental and Occupational Health and Safety, Institute of Public Health, College of Medicine and Health Sciences, University of Gondar, Gondar, Ethiopia

**Keywords:** Low birth weight, Preterm birth, Still birth, Northwest Ethiopia

## Abstract

**Background:**

Adverse birth outcomes are major public health problems in developing countries. Data, though scarce in developing countries including Ethiopia, on adverse birth outcomes and the risk factors are important for planning maternal and child health care services. Hence, this study aimed to determine the prevalence and associated factors of adverse birth outcomes among deliveries at Gondar University Hospital, Northwest Ethiopia.

**Methods:**

Institution based cross-sectional study was conducted in February 2013 at Gondar University Hospital. Data were collected by face-to-face interview of 490 women after verbal informed consent using a pretested and structured questionnaire. Gestational age was determined based on the last normal menstrual period. Birth weight was measured following standards. Multiple logistic regressions were fitted and odds ratios with their 95% confidence interval were computed to identify associated factors.

**Results:**

The mean age of women was 26.2 (±5.2 SD) years. HIV infection among laboring women was 4.8%. About 23% of women had adverse birth outcomes (14.3% preterm, 11.2% low birth weight and 7.1% still births). Women having history of either preterm delivery or small baby (AOR: 3.1, 95% CI 1.1- 8.4) were more likely to have preterm births. Similarly, history of delivering preterm or small baby (AOR: 8.4, 95% CI 2.4- 29.4), preterm birth (AOR: 5.5, 95% CI 2.6- 11.6) and hypertension (AOR: 5.8, 95% CI 1.8- 19.6) were associated factors with low birth weight. Ante partum haemorrhage (AOR: 8.43, 95% CI 1.28- 55.34), hypertension (AOR: 9.5, 95% CI 2.1-44.3), history of perinatal death (AOR: 13.9, 95% CI 3.3- 58.5) and lack of antenatal care follow up (AOR: 9.7, 95% CI 2.7 - 35.8) were significantly associated with still birth.

**Conclusions:**

Prevalence of adverse birth outcomes (still birth, preterm birth and low birth weight) were high and still a major public health problem in the area. Histories of perinatal death, delivering preterm or small baby, ante partum hemorrhage, lack of ante natal care follow up and hypertension were associated factors with adverse birth outcomes. Thus, further enhancements of ante natal and maternal care and early screening for hypertension are recommended.

## Background

Adverse birth outcomes- such as prematurity, low birth weight and birth defects- represent significant problems in both developing and developed countries. Each year, about 15 million babies in the world, more than one in 10 births, are born too prematurely. More than one million of those babies die shortly after birth; countless others suffer from lifelong physical, neurological, or educational disabilities, often at great cost to families and societies [1,2]. Complications of preterm birth are the leading direct causes of neonatal mortality and account for an estimated 27% of neonatal deaths. This comes to almost four million neonatal deaths every year [3].

From a global standpoint, the prevalence rate of preterm birth varies from 47.5 to 137 per 1000 live births. Extreme parity, a previous history of preterm birth or abortion, younger maternal age, inadequacy of prenatal care, reported hypertension, antepartum hemorrhage, premature rupture of fetal membranes and induced labor are significant determinants of preterm birth [4-6].

Worldwide stillbirth rate has declined by 14%, from 22.1 stillbirths per 1000 births in 1995 to 18.9 stillbirths per 1000 births in 2009. But in the African region, there was only an annual decline of less than 1%. The stillbirth rate for developed countries is estimated between 4.2 and 6.8 per 1000 births, whereas for the developing world, the estimate ranges from 20 to 32 per 1000 births. Two thirds of all stillbirths occur in just two regions: South-East Asia and Africa [7,8]. In sub-Saharan Africa, an estimated 900,000 babies die as stillbirths. It is estimated that babies who die before the onset of labor, or ante partum stillbirths, account for two-thirds of all stillbirths in countries where the mortality rate is greater than 22 per 1,000 births [9]. From previous studies, preterm birth, increasing maternal age, history of stillbirth, reported hypertension, extremes of neonatal birth weight, cesarean delivery, operative vaginal delivery, and assisted breech delivery were all significantly associated with stillbirth [10-12].

Low birth weight (LBW) is closely associated with increased fetal and neonatal mortality, morbidity, and impaired growth and cognitive development. It also leads to chronic diseases later in life. Worldwide, more than twenty million infants (representing 15.5% of all births, 95.6% of whom in developing countries) are born LBW [13]. Several studies reported that prematurity, previous histories of adverse birth outcomes, maternal age, anemia and inadequate food intake during pregnancy, and lack of antenatal care (ANC) follow up were associated factors of LBW [14-18].

In Ethiopia, adverse outcome of pregnancy are still major public health problems [18,19]. The achievement of Millennium Development Goal (MDG) 4 is strongly influenced by progress in reducing neonatal deaths. Since preterm birth is the leading cause of neonatal mortality progress of MDG-4 is dependent on achieving high coverage of evidence-based interventions that halt preterm deliveries and improve survival for preterm newborns [7]. In general, epidemiological data on the magnitude and risk factors of adverse birth outcomes are important for planning maternal and child health care services in developing countries. Hence, this study aimed to determine prevalence and associated factors of adverse birth outcomes of pregnancy at a teaching referral hospital in Northwest Ethiopia.

## Methods

A hospital based cross-sectional study was conducted at the maternity wards of Gondar University hospital in February 2013. This hospital is the only tertiary hospital located in the historical city of Gondar. It serves for over five million people residing in urban and rural parts of northern and northwestern Ethiopia. On average, there are about 20 deliveries everyday in this hospital. The study included 490 laboring women selected consecutively during the data collection period. This study included all women who gave birth throughout the day and night during the one month study period.

Low birth weight was defined as a birth weight below 2500 grams (5.5 pounds). If the baby was born before 37 completed weeks of gestation but after 28 weeks of gestation, it was considered as preterm. Stillbirth was defined as the birth of an infant that has died in the womb or during intra partum after 28 weeks of gestation. Gestational age was calculated based on the last normal menstrual period (LNMP). When LNMP-based gestational age was unknown, we relied on ultrasonography measures and nine women were excluded from the analysis since gestational age was not determined using either method. Birth weight was measured for each new born within an hour of birth using a calibrated weight scale.

Data were collected using a combination of a structured questionnaire and measurements of weight of the new born by eight midwives who were trained for this purpose. The questionnaire was structured into four logical sections (socio demographic characteristics, obstetrics related factors; medical history and birth outcomes assessment). Data were entered into EPI Info version 3.5.3 and exported to SPSS version 20 for analysis. Descriptive statistics like frequencies and cross tabulations were performed. Multiple logistic regressions were fitted for the three major adverse birth outcomes separately and odds ratio (OR) with their 95% confidence interval (95% CI) were calculated to identify associated factors of adverse birth outcomes. Variables with p-values ≤ 0.2 in bivarate analysis were remained in the model as potential confounders for the next level analysis. The Hosmer -Lemeshow goodness-of-fit statistic was used to check if the necessary assumptions for multiple logistic regressions were fulfilled and the model had p-value >0.05 which proved the model was good.

Ethical clearance was obtained from the University of Gondar Institutional Review Board. Permission letter was also obtained from hospital administration office. Data were collected after informed verbal consent was obtained and after the women were stabilized and ready to be discharged. Confidentiality of the information was assured from all the data collectors and investigators sides. The questionnaire was administered anonymously, locked with keys (hard copy) and password protected (soft copy). Those who had adverse birth outcomes were linked for additional services (i.e. preterm and LBW births were linked to neonatology ward, still births were reassured and advised to have ANC follow up when they get pregnant again).

## Results

### Socio demographic characteristics

A total of 481 laboring women were included in this study. The mean age was 26.2 (±5.2 SD) years. Majority were Orthodox Christians (83.8%), and Amhara (97.1%) ethnics. Most (92.7%) were married, and more than half (55.1%) were housewives. About one quarter (24.9%) of the participants attended secondary education. The mean age at first marriage was 19.3 (±3.7SD) years (Table 1).

**Table 1:** Socio-demographic characteristics of respondents’, Gondar University Hospital, Northwest Ethiopia, February 2013 (n = 481)

**Characteristics**	**Frequency**	**Percent**
Residence		
Urban	359	74.6
Rural	122	25.4
Age		
<20	27	5.6
20-34	404	84.0
35^+^	50	10.4
Marital status		
Single	32	6.7
Married	446	92.7
Others^a^	3	0.6
Education level		
No formal education	157	32.6
Primary level	113	23.5
Secondary level	118	24.5
Tertiary level	93	19.3
Occupation		
Farmer	58	12.1
Housewife	265	55.1
Merchant	52	10.8
Government employee	84	17.5
Others^b^	22	4.6
Religion		
Orthodox	403	83.8
Muslim	54	11.2
Others^c^	24	5.0
Ethnicity		
Amhara	467	97.1
Tigrie	14	2.9
Age at 1^st^ marriage		
Under 18	127	26.4
≥18	354	73.6
Family size		
≤ 5	414	86.1
>5	67	13.9

^a^*mainly divorced*, ^b^*jobless or daily laborer*, ^c^*mainly protestant*.

### Obstetrics related characteristics

Majority of respondents (86.3%) had ante natal care (ANC) follow and 17.8% had started their follow up during the first trimester of pregnancy. About three fifth (57.8%) of them had at least 4 ANC visits. Nearly three quarters (73.2%) were using modern contraceptives prior to the current pregnancy, 70% injectible methods. Similarly, nearly three quarter (73%) of the respondents had nutritional counseling and about 72% had additional diet during the current pregnancy.

Most labors (91.1%) were spontaneously initiated. The mean duration of labor was 9.4 (±5.9 SD) hours. About 81% of current deliveries were spontaneous vaginal deliveries (SVD) and or assisted vaginal deliveries and 13.9% by caesarian section (CS).

Historically, 6.7% of women reported pre natal death in the preceding birth. The pregnancies of most (86.3%) women were planned and wanted. More than one fifth (22.5%) of participants had premature rapture of membrane (PROM) in the current delivery (Table 2).

**Table 2:** Obstetrics related characteristics of respondents’, Gondar University Hospital, Northwest Ethiopia, February 2013

**Characteristics**	**Frequency**	**Percent**
ANC follow up status		
Yes	415	86.3
No	66	13.7
No of ANC visits		
1 times	10	2.1
2-3 times	127	26.4
≥4 times	278	57.8
Time of 1^st^ ANC Visit		
1^st^ trimester	74	17.8
2^nd^ trimester	306	73.7
3^rd^ trimester	35	8.5
Modern contraceptive use prior to current pregnancy		
Yes	352	73.2
No	129	26.8
Types of contraceptive used		
Injectible	246	69.9
Pills	91	25.9
Others^a^	15	4.2
Dietary counseling during pregnancy		
Yes	351	73.0
No	130	27.0
Additional diet during pregnancy		
Yes	347	72.1
No	134	27.9
Parity		
Premipara	203	42.2
Multipara	278	57.8
Mode of delivery		
SVD	390	81.1
Instrumental delivery	24	5.0
CS	67	13.9
Labour status		
Spontaneous	438	91.1
Induced	43	8.9
Labor duration		
≤ 9.4 hours	253	52.6
>9.4 hours	228	47.4
Poor obstetrics history		
None	410	85.2
Perinatal death	23	6.7
Abortion	33	4.6
Preterm/small baby	17	3.5
PROM in this pregnancy		
Yes	108	22.5
No	373	77.5
Congenital malformation		
Yes	20	4.2
No	461	95.8
Pregnancy status		
Planed and wanted	415	86.3
Unplanned but wanted	45	9.3
Unplanned and unwanted	21	4.4
Birth space in years		
<3	105	37.2
3-4	101	35.8
5+	76	27.0

^a^*mainly Norplant and intra uterine device (IUCD)*.

### Medical and other obstetrics related characteristics

In this study, 11% of participants had history of fever of 2 weeks or more during current pregnancy and 10% had been diagnosed anemic during the current pregnancy. Majority of the respondents (99.0%) were screened for HIV and about 5% were sero-positive (Table 3).

**Table 3:** Medical and other obstetrics related characteristics of respondents’, Gondar University Hospital, Northwest Ethiopia, February 2013

**Characteristics**	**Frequency**	**Percent**
Adverse birth outcome (at least one)		
Yes	109	22.7
No	372	77.3
All 3 key adverse birth outcomes		
Yes	16	3.3
No	465	96.7
Fever (≥ 2 weeks)		
Yes	54	11.2
No	427	88.8
Medical illness		
Yes	118	24.5
No	363	75.5
Types of medical illness		
Anemia	49	10.2
UTI	22	4.6
Malaria	13	2.7
HIV/AIDS	23	4.8
Others	11	2.3
Hypertension		
Yes	19	4.0
No	462	96.0
Ante partum hemorrhage		
Yes	12	2.5
No	469	97.5
Post partum hemorrhage		
Yes	16	3.3
No	465	96.7
HIV screening status		
Yes	476	99.0
No	5	1.0
HIV test result (n = 476)		
Positive	23	4.8
Negative	453	95.2
ART status (n = 23)		
Started	18	78.3
None	5	21.7
Physical harassment		
Yes	8	1.7
No	473	98.3
Time to reach nearby health facility		
≤30 minutes	231	48.0
>30 minutes	250	52.0

### Prevalence and associated factors of still birth

The over prevalence of still birth was 7.1%. As shown in the multivariate analysis model, risk factors like preterm birth, low birth weight, ante partum hemorrhage (APH), hypertension, history of perinatal death, lack of ANC follow up and large family size (>5) were significantly and independently associated with still birth (Table 4).

**Table 4:** Logistic regression analysis of factors associated with still birth among deliveries in Gondar University Hospital, Northwest Ethiopia (n = 481), February 2013

**Variables**	**Still birth**		**Crude OR**	**Adjusted OR**
	**Yes**	**No**	**(95% CI)**	**(95% CI)**
Residence				
Urban	17	342	1	1
Rural	17	105	3.26 (1.61-6.61)	1.64 (1.29- 8.24)
Age (years)				
<20	6	21	1	1
20-34	25	379	0.23 (0.09 – 0.62)	0.17 (0.02- 1.21)
35^+^	3	48	0.22 (0.05 -0.98)	0.02 (0.00- 0.32)
Occupation				
Farmer	14	44	1	1
House wife	12	253	0.15 (0.06-0.34)	0.19 (0.05-0.79 )
Merchant	3	49	0.19 (0.05-0.71)	0.35 (0.04- 2.94)
Government employee	2	82	0.08 (0.02-0.35)	0.06 (0.01- 0.89)
Others*	3	19	0.50 (0.13-1.93)	0.62 (0.07- 5.78)
Family size				
≤ 5	25	389	1	1
>5	9	58	2.41 (1.10-5.43)	5.46 (1.46- 20.40)
ANC follow up status				
Yes	18	397	1	1
No	16	50	7.11 (3.39-14.72)	9.74 (2.65- 35.77)
Birth weight				
LBW	21	33	20.27 (9.32-44.10)	18.21 (6.06 - 55.34)
Normal	13	414	1	1
APH				
Yes	5	7	10.84 (3.24-36.26)	8.43 (1.28- 55.34)
No	29	440	1	1
Hypertension				
Yes	6	13	7.15 (2.53-20.24)	9.53 (2.05-44.33)
No	28	434	1	1
History of perinatal death				
Yes	8	17	7.78 (3.10-19.70)	13.90 (3.30- 58.53)
No	28	430	1	1
Gestational age				
Preterm	17	52	7.6 (3.65 – 15.79)	4.47 (1.39 – 14.32)
Term	17	395	1	1

*were mainly jobless or student.

### Prevalence and associated factors of preterm birth

Nearly one in seven births (14.3%) was found to be preterm birth. The mean gestational age was 37.1 (±1.7 SD) weeks. Women who had history of either preterm delivery or low birth weight (AOR: 3.10, 95% CI 1.12- 8.36) were more like to have preterm birth than their counter parts. On the other hand, hypertension was significant (COR: 2.92, 95% CI 1.10-7.97) in the bivariate analysis but turned out insignificant in the multivariate analysis.

### Prevalence and associated factors of low birth weight (LBW)

In this study, 11.2% of deliveries were found to be LBW. The mean neonatal birth weight was 2977.7 (±573.5 SD) grams. In multivariate analysis, history of preterm delivery/or small baby (AOR: 8.40 95% CI 2.40- 29.40), preterm delivery (AOR: 5.51 95% CI 2.61- 11.62) and hypertension (AOR: 5.84 95% CI 1.75- 19.55) remained significantly and independently associated with LBW (Table 5).

**Table 5:** Logistic regression analysis of factors associated with LBW among deliveries in Gondar University Hospital, Northwest Ethiopia (n = 481), February 2013

**Characteristics**	**LBW**		**Crude OR**	**Adjusted OR**
	**Yes**	**No**	**(95% CI)**	**(95% CI)**
Residence				
Urban	36	323	1	1
Rural	18	104	1.55 (0.85- 2.85)	1. 13 (0.45- 2.18)
Pregnancy type				
Singleton	49	419	1	1
Multiple	5	8	5.34 (1.68- 16.98)	2. 26 (.34- 15.10)
ANC follow up status				
Yes	42	373	1	1
No	12	54	1.94 (0.98- 3.98)	0.98 (.35- 2.43 )
No of ANC visits				
1 times	2	9	1.09 (0.13- 8.84)	0.35 (0.02- 7.25)
2-3 times	15	113	1.30 (0.67- 2.55)	0.89 (0.37- 2.12)
≥4 times	25	252	1	1
Dietary counseling				
Yes	34	317	1	1
No	20	110	1.70 (0.94-3.07)	1. 75 (0.75 - 4.10)
Parity				
Premipara	25	178	1.21 (0.68- 2.13)	1.29 (0.62- 2.69)
Multipara	29	249	1	1
PIH				
Yes	7	12	5.15 (1.93- 13.72)	5.84 (1.75- 19.55)
No	47	415	1	1
APH				
Yes	4	8	4.19 (1.22- 14.42)	2.49 (0.43- 14.29)
No	50	419	1	1
Anemia				
Yes	12	56	1.89 (.94- 3.81)	1.49 (0.79- 4.74)
No	42	371	1	1
History of preterm/small baby				
Yes	9	10	8.34 (3.22- 21.60)	8.40 (2.40- 29.40)
No	45	417	1	1
Gestational age				
Preterm	30	382	6.79 (3.66- 12.62)	5.51 (2.61- 11.62)
Term	24	45	1	1

## Discussion

In this study, we assessed the prevalence and associated factors of adverse birth outcomes (still birth, preterm birth, and low birth weight) among deliveries at Gondar University hospital. The prevalence of still birth was 71 per 1,000 total births. This prevalence is higher than would be expected from a community based study since the study center is a tertiary hospital managing referrals from health centers and district hospitals. It is also higher than the previous reports from Nigeria, Zambia and a systemic review for sub-Saharan African studies where the prevalence of still birth ranged from 21-33/1,000 total births [10,12,20]. Methodological and socio-economic variations explain differences in adverse birth outcomes [21]. It could be also partially explained by variation in the study subjects, for instance, the report from Zambia was limited to urban residents unlike the current study which included rural residents too. It is also higher than the 2009 WHO African regional estimates of stillbirth rates (28.1/1,000 total births) [7]. However, this was a hospital based cross-sectional study unlike the WHO African regional estimates of stillbirth rates for communities. Most normal deliveries take place in health centers while more complicated ones are referred to the tertiary hospital contributing to higher rates of adverse birth outcomes at referral hospitals. Moreover, women who experienced obstetric complications are likely to show up to health facilities and may get referred to hospitals; higher rates of adverse birth outcomes may exist at referral hospitals. A research from Southwest Ethiopia [22] reported a higher prevalence than our study (119/1,000 live births), however it included all deaths that occurred until discharge.

This study also revealed that nearly one in seven births (14.3%) was preterm. This is lower than a previous finding from Uganda among HIV-positive rural mothers (17.7%) [23]. This variation may be due to difference in populations studied, as participants of the current study were predominantly urban residents (75.6%), and HIV-negative (95.2%). However, it was higher than reports from China (4.75%), Nigeria (12%) and Brazil (13.7%) [4-6]. This difference may be due to methodological and population variation on top of the socio economic and set up differences.

The prevalence of LBW in this study was 11.2%. This is lower than a previous study in the same hospital (17.1%) [18]. This might be due to seasonal variations in birth weight [21]. Similarly, this finding is lower than reports from southwest Ethiopia (22.5%), west Bengal (28.8%) and Ethiopian Demographic Health Survey (EDHS) 2011 report (28%) [14,16,24]. This high discrepancy is mainly due to the methodological variations. In this study, we measured birth weights with standard procedures and instruments within an hour of birth. However, the EDHS report was mainly based on subjective maternal assessment of birth weights (as normal, big, small or very small). The current study was also limited to the tertiary hospital and was purely cross-sectional. However, aforementioned studies were community based. It is an established fact that socio-economic, racial/ethnic individual and contextual differences determine birth weight [25,26].

In multivariate analysis, women who did not have ANC follow up were more likely to have stillbirth. During ANC follow up women will have access to information related to nutrition and danger signs of pregnancy. Regular ANC follow up will also help a pregnant woman seek early treatment for her potential pregnancy related problems but if failed to showed up for ANC, she will be disadvantaged. Additionally, women who did not have ANC follow up were mostly illiterate (60.3%) and hence may not have good healthcare seeking behaviors. This finding is in line with previous studies in Africa [18,20,22]. Similarly, women who had hypertension during the current pregnancy were six times more likely end up with stillbirths. This is mainly because of placental insufficiency as evidenced in previous reports [11,20]. Gestational age was another predictor of stillbirth; those preterm newborns were about six folds more likely to be born as a stillbirth. Preterm newborns are usually immature and fail to survive till birth. This finding supports other previous findings from sub-Saharan Africa [10,27]. Ante-partum hemorrhage during the current pregnancy led to stillbirth. Bleeding during pregnancy is one of the etiologies of anemia leading to intra-uterine oxygen inadequacy [10].

Furthermore, women having history of perinatal death in the preceding births were at higher risk of having stillbirths. Most poor obstetrics histories are recurrent. LBW was also found to be associated with stillbirth in this study. Similar to other studies [12,27], LBW babies were most likely born as stillbirth. In general, most stillbirths would have been prevented through antenatal follow up and its interventions. In this particular study, similar to a Brazilian report [6], previous history of preterm/small baby delivery was associated with preterm birth.

Owing to the fewer number of cases the significant association in the crude analysis between hypertension and preterm birth was insignificant after adjustment. In the adjusted analysis, LBW was more common in women who had hypertension, had previous history of preterm and/or small baby deliveries and among preterm newborns. Hypertension is one of the causes of preterm deliveries and immature newborns are more likely to be LBW [28]. It is possible to early identify women with hypertension in their ANC follow up and take appropriate measures. In a hospital based study in Rwanda, LBW was more common in those women who had history of previous preterm birth [29]. Similarly, in a hospital based cross-sectional study in southwest Ethiopia, preterm delivery was also significantly associated with LBW [16].

This study shares the limitations of cross-sectional studies and hence may not be possible to establish temporal relationship between adverse birth outcomes and explanatory variables. Besides, as the study was in a referral hospital, it may not show the real picture of these adverse birth outcomes in the area. Another limitation is possible recall bias while determining the gestational age.

## Conclusions

Adverse birth outcomes (still birth, preterm birth and LBW) are still major public health problems in this area.

Histories of perinatal death, preterm birth and/or small baby, ante partum hemorrhage, absence of ANC follow up and hypertension were associated with adverse birth outcomes. Hence, further enhancements of antenatal and maternal care as well as early screening for hypertension are important recommendations. We also recommend a more representative community based study.

## Competing interests

The authors declare that they have no conflict of interests.

## Authors’ contributions

AA wrote the proposal, participated in data collection, analyzed the data and drafted the paper. BM, TA, LG and BD approved the proposal with some revisions, participated in data collection, analysis and manuscript writing. All authors read and approved the final manuscript.

## Pre-publication history

The pre-publication history for this paper can be accessed here:

http://www.biomedcentral.com/1471-2393/14/90/prepub
